# Cloth Face Coverings for Use as Facemasks During the Coronavirus (SARS-CoV-2) Pandemic: What Science and Experience Have Taught Us

**DOI:** 10.1017/dmp.2020.354

**Published:** 2020-09-14

**Authors:** Raymond J. Roberge, Marc R. Roberge

**Affiliations:** Consultant, respiratory protective equipment, Pittsburgh, Pennsylvania; Attending physician, University of Pittsburgh Medical Center East, Monroeville, Pennsylvania

**Keywords:** cloth face coverings, efficacy, infection control, pandemic, surgical masks

## Abstract

The current coronavirus (severe acute respiratory syndrome coronavirus 2 [SARS-CoV-2]) pandemic has resulted in severe shortages of personal protective equipment, including respiratory protective equipment, such as N95 respirators. This has led some government agencies to suggest the use of cloth face coverings (CFCs) by health-care providers and the general public as a last resort when standard respiratory protective equipment is unavailable. Although such coverings have been in use for over a century and have found widespread use during some previous pandemics, research data are relatively scant for the protective value of this measure. This article, a literature review, explores the development of CFCs and reviews available scientific research regarding the efficacy of this intervention as a preventive measure in the spread of airborne infectious diseases

The current coronavirus (severe acute respiratory syndrome coronavirus 2 [SARS-CoV-2]) pandemic has resulted in worldwide shortages of personal protective equipment, especially respiratory protective equipment, such as N95 respirators and surgical facemasks (SMs). This shortage has resulted in a precarious dilemma for many individuals, including health-care providers (HCPs) administering frontline care to pandemic victims and other caregivers of infected persons. Inadequately protected HCPs and caregivers risk being infected themselves and become a contact source for spreading the coronavirus. If HCPs are not suitably protected, the health-care system can no longer function at the requisite level necessary to address the pandemic. Attempts to increase the manufacture and supply of respirators and SM during a pandemic are necessarily time-dependent due to such issues as disrupted supply lines, decreased availability of manufacturing materials, re-tooling issues, and availability of healthy industrial workers.

This lack of commercially available respiratory protective equipment has spawned the idea of the fabrication and use of homemade cloth face coverings (CFCs) as a temporizing measure while awaiting the manufacture and delivery of commercially manufactured products. The use of CFCs by the public could also free up limited supplies of SMs and respirators for HCPs. The recent Centers for Disease Control and Prevention (CDC) recommendation that “*cloth face coverings be worn in public settings where other social distancing measures are difficult to maintain (eg, grocery stores and pharmacies), especially in areas of significant community-based transmission* ^1^” further highlights the potential for widespread use of this controversial measure. The difficulties inherent in studying the effect of CFC use on large populations have resulted in widespread dispersal of information regarding this intervention that is largely founded on assumptions. Based on the results of a literature search and their prior research experience with the National Institute for Occupational Safety and Health’s National Personal Protective Technology Laboratory, a government agency that tests and certifies respiratory protective equipment, the authors provide an overview of the development of CFCs and their functional capabilities compared with commercially available SMs (the type of commercial mask most likely to be used by the general public^2^).

## METHODS

An English-language literature search was conducted from March 27, 2020, to April 6, 2020, using the PubMed database for the search terms cloth mask, cloth facemask, cloth face coverings, homemade mask, homemade face covering, filtration, source control, and efficacy. Additionally, an Internet search with the Google engine was performed using the search terms cloth masks, homemade masks, filtration, efficacy, government recommendations, and randomized trials. Articles were deemed pertinent by title and abstract review of both authors; culled articles were then read fully by both authors for agreement on inclusion. Bibliographies of culled articles were scanned for possible additional references.

## RESULTS

**FIGURE 1:**
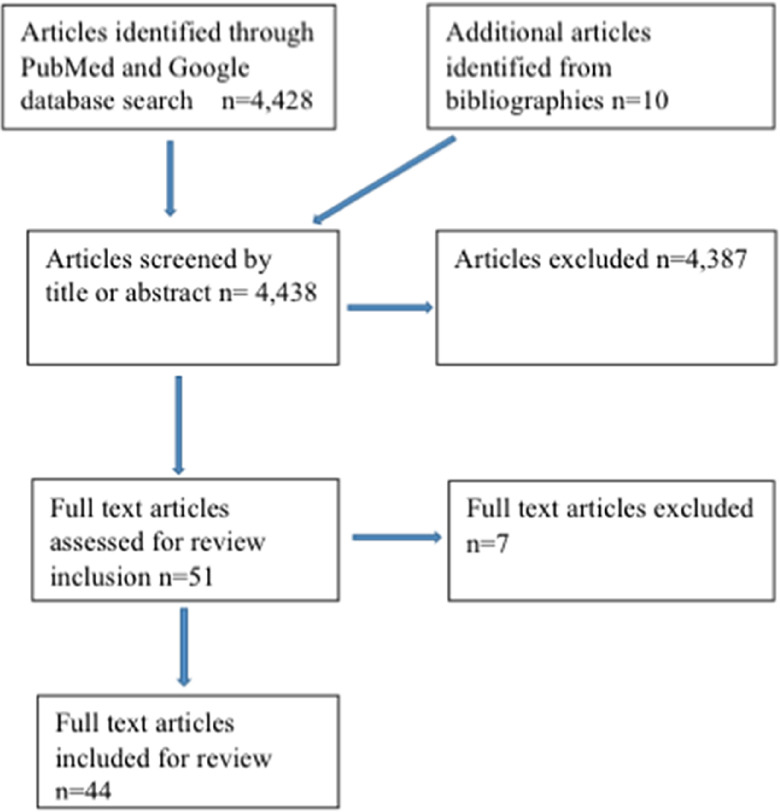
Literature search components.

A total of 4428 published peer-reviewed articles was retrieved, of which 41 articles were determined to be related to the topic after review of titles and abstracts by both authors. Another 10 peer-reviewed research publications were gleaned from the bibliographies of the aforementioned 41 publications for a total of 51 articles considered for possible inclusion. After a full read of all 51 articles, 7 were excluded, leaving a total of 44 articles to serve as the basis for this review. Additionally, 1 government review of respiratory protection and 13 Web sites (11 government sites, 1 private industry site, 1 university site) related to the topic were retrieved and incorporated into the report (Figure 1). The results of the literature review are summarized by category, as follows:

### Evolution of CFCs as Surgical Facemasks

The concept of a facemask was initially introduced in 1897 by the Polish surgeon Mikulicz-Radecki,^3^ based on the theory of the infectious nature of exhaled respiratory droplets in the transmission of tuberculosis, as proposed by the German physician Flugge.^4^ The first efforts at fabricating facemasks used the use of multiple layers of gauze applied over the mouth of surgical staff in an attempt to protect sterile surgical fields and surgical patients from aerosol droplets generated by staff, as well as to protect the surgical staff from blood and other body fluids expelled during surgery.^5^ In 1905, Alice Hamilton, a physician at the Memorial Institute for Infectious Diseases (Chicago), proposed the use of facemasks in surgery to prevent surgical infections after her studies demonstrated that streptococci were expelled from the mouth of surgeons by “*invisible sputum from coughing, speaking, whispering, crying or breathing forcibly from the mouth*”.^6^ In 1918, Capps demonstrated the utility of facemask use by staff and patients alike in suppressing the spread of measles and scarlet fever in hospital wards.^7^ The use of facemasks by surgical staff became routine, although not universal, by 1920,^8^ and over the past several decades has been used by medical staff and the general public as protection from airborne large respiratory aerosol droplets.^9^

### Descriptive Characteristics of SMs and CFCs

SM are Food and Drug Administration (FDA) approved medical devices that provide barrier protection against large droplets (>5 µm) expelled from the respiratory tract of the wearer (source control) and protect the wearer’s mouth, nose, and part of the facial skin from exposure to large aerosol splashes, sprays, and splatter of body fluids from other sources. SMs are not considered respirators (eg, N95 respirators) because they cannot filter small airborne particles, vapors, or gases. Moreover, because of their loose fit, as opposed to the tight fit of respirators, they do not prevent air leakage around the edges of the mask during inhalation.^10-12^ SM are disposable and generally consist of 3-4 layers of nonwoven bonded fabric, often overlapping 2 layers of filters, the combination of which is capable of filtering out particles that are ≥1 µm in diameter, thereby trapping bacteria of that size or larger.^13^ The FDA does not test SMs, but relies on test data supplied by manufacturers (fluid resistance, efficiency for particulate filtration [using 0.1 µm latex spheres], and bacterial filtration [using 3.0 µm particles containing *Staphyococcus aureus* bacteria], differential pressure [pressure drop across a SM], and flammability).^12,14^ FDA approval is granted if the manufacturer-supplied data is equivalent or better than currently approved SM.^14^

CFC were initially made for surgical personnel and were composed of multiple layers of gauze that covered the mouth.^15^ Gauze likely was used because it was readily available in the health-care setting given its use as bandages, wound dressings, and surgical sponges. During the Manchurian plague (1910-1911), CFCs were made of 2 layers of gauze encasing a 4- × 6-inch piece of absorbent cotton.^16^ Cotton CFCs were in widespread use by the public and HCPs during the 1918 Spanish influenza and the use of cotton and gauze CFC continued through the first half of the 20th century until the introduction of disposable facemasks in the 1950s and 1960s.^17^ Over the ensuing years, several other common fabrics have been used for CFCs, including polyester, cotton/polyester combinations, fleece, and linen.^18^ Although there are no universal guidelines for fabricating CFCs, suggestions from fabric experts and health-care agencies highlight the need to preferentially use breathable, tight-weave cotton fabrics (double layered, if possible), ideally with a moisture-wicking (eg, microfleece) middle layer and avoidance of knit fabrics (create holes when they stretch) and woolens (may irritate the skin of sensitive individuals).^19-21^ Fabrics should be prewashed in hot water to disinfect and preshrunk before CFC construction^21^; shrinkage through repeated washing of gauze has previously been shown to increase the protective nature of the fabric.^22^

### Protection Afforded by Cloth Face Coverings

#### a) Protection Factors

A respirator’s ability to protect is greatly dependent on leakage at the face/respirator interface and through its filter and exhalation valve (if so equipped), with the fit of the respirator to the face as the primary component. Quantification of the protection afforded by tight-fitting respirators (eg, N95 respirators) can be obtained by Occupational Safety and Health Administration (OSHA) quantitative fit testing that measures the concentration of airborne challenge aerosol particles in the environment and particles within the space between the wearer’s face and the inner surface of the respirator (deadspace) during various activities.^23^ The ratio of the 2 concentrations is termed the fit factor and a minimum passing score of ≥100 is derived from the N95 respirator’s assigned protection factor of 10 (the expected workplace protection afforded by a respirator or class of respirators indicating that no more than one-tenth of contaminants will leak into the respirator) multiplied by a safety factor of 10 (that compensates for the likely better fit of a respirator within controlled laboratory settings compared with the work environment).^24^ A score of 100 from a quantitative fit test indicates that ≤1% of challenge aerosols enter the deadspace of the respirator. Although the loose fit of SMs and CFCs makes quantitative fit testing unwarranted, a small number of research studies have fit tested SMs and CFCs to determine what protection may be afforded the wearer.

Three human studies of SMs have demonstrated protection factors ranging from 1.3 to 6.5, considerably less than a properly fitting N95 respirator.^25-27^ Another human study reported protection factors ranging from 2 to 12 for SMs.^28^ Dato et al.^29^ tested a CFC of 2-ply heavyweight tee-shirts in 8 inner layers arranged in different orientations and having 3 sets of ties on 3 subjects and reported nonpassing OSHA fit factors 67, 13, and 17 for 3 individuals tested, indicating significant penetration through the CFC material, leakage around the seal of the CFC, or both, that indicate a low protection factor.^29^ A study of 20 subjects testing 4 models of SM reported fit factors ranging from 2.5 to 9.6.^30^ van der Sande et al.^27^ reported protection factors ranging from 2.2 to 3.2 for adults and 1.9-2.2 for children wearing CFCs made from tea cloth. Davies et al.^2^ noted that CFCs fabricated from tee-shirts provided a median fit factor (2) less than half that of SMs (5) in 21 subjects (Table 1).

**TABLE 1:** Protection factors and fit factors of surgical masks (SM) and cloth face coverings (CFC)

Reference	Protective Factor		Fit Factor	
	SM	CFC	SM	CFC
Kournikakis et al.[Bibr r25]	1.6-5.6			
Lee et al.[Bibr r26]	2.4 (mean)		1.7-2.5	
Gwan et al.[Bibr r28]	2-12			
van der Sande[Bibr r27]			4.1-5.3*	2.2-3.2*
			3.2-4.9§	1.9.-2.2§
Davies et al.[Bibr r2]			5 (median)	2 (median)
Oberg et al.[Bibr r30]			2.5-9.6	
Dato et al.[Bibr r29]				13-67

*Adults. §Children.

#### b) Viral Penetration

Nonhuman studies of SM and CFC materials have examined the penetration of viral aerosols or aerosols that are representative of viruses (20-400 nm). A study of 2 models of SM from the same manufacturer, challenged with MS2 virus, noted widely different viral penetrations of 20.5% and 84.5% at a flow rate of 85 L/min.^31^ Another study evaluating 7 different models of SMs to direct challenge with influenza aerosol reported a general reduction in exposure of 1 log or less.^28^ Rengasamy et al.^18^ reported that the penetration levels for 400 nm aerosol particles at a standard face velocity for testing filters (5.5 cm s^-1^) reached 73-87% for commercial cloth masks; the 3 sweatshirt and tee-shirt models were in the 30-61% and 56-79% ranges, respectively, for 20-nm size particles and increased to 80-93% and 89-97% as particles reached 1000 nm. Another study^2^ noted that SM had maximum filtration efficiency (ability of a respirator filter to prevent the passage of aerosol particles) of 89.5% for viral-sized particles and CFC of various fabrics (100% cotton tee-shirt, scarf, tea towel, pillow case, cotton/polyester mix, linen, silk) had efficiencies of 48.8-70.2%. Lee et al.^26^ reported that 3 models of SMs had the most penetrating particle sizes ranging from 20 to 200 nm, the size range that includes SARS-causing coronaviruses and influenza viruses.

Aerosol testing of 2 models of cloth masks against polystyrene particles of diameters of 1.0 nm and 2.5 nm, and particles with virus-sized dimensions (30, 100, and 500 nm), indicated filtration ranging from 15 to 57% that decreased with decreasing particle size and was inferior to results for SM.^32^ MacIntyre et al.^33^ reported aerosol penetrations of 97% and 44%, respectively, for cloth face masks commonly used in Asia and medical masks. Testing handkerchiefs of cotton, gauze, or towel material to sodium chloride aerosols of 75 ± 25 nm count median diameter indicated penetrations of 97% using an automated filter tester at a flow rate of 95 L/min (normal breathing flow rate is 15-30 L/min)^34^ (Table 2).

**TABLE 2:** Viral Penetration Studies of SMs and CFCs

	SMs	
Reference	Study Type	Findings
Balazy et al.^31^	Nonhuman	Viral penetrations of 20% and 84.5% for 2 models of SM from same manufacturer (flow rate 85 L/min)
Gawn et al.^28^	Human	Reduction in viral exposure influenza aerosols <1 for 7 models of SM
Lee et al.^26^	Nonhuman	Most penetrating particle size for 3 models of SM was 20-200 nm
Davies et al.^2^	Human	89.5% filtration efficiency against virus-sized particles for SM
MacIntyre et al.^33^	Nonhuman	97% viral penetration through SM
	**CFCs**	
Jung et al.^34^	Nonhuman	97% penetrations of virus-sized particles for CFC
Rengasamy et al.^18^	Nonhuman	Particle (20 nm) penetrations of 30%-61% for sweatshirt CFC and 56%-79% for tee shirt CFC
Davies et al.^2^	Human	48.8-72.2% filter efficiency of virus particles for CFC
Shakya et al.^32^	Nonhuman	Filtration efficacy of 15-57% for 4 types of CFC
MacIntyre et al.^33^	Nonhuman	Filter penetrations of 97% for CFC

#### c) Bacterial Penetration

A human study testing the efficacy of various fabrics (eg, bath towel, cotton shirt, handkerchief) noted filtration efficiencies of 28-73% against *Bacillus globigii* aerosols (2000 nm).^35^ A mannequin study, using aerosolized droplet nuclei in the range of bacterial sizes (1.0-2.5 µm), noted filtration efficiencies of 33.1% for SM and 11.3% for bandanas.^36^ Another study^2^, using a closed-circuit filter testing system, demonstrated bacterial filtration efficiencies of 96.3% for SM and 60-83.2% for various common fabrics used for CFC (100% cotton tee-shirt, scarf, tea towel, pillow case, cotton mix, linen, silk). Research using green cotton fabric used for hospital operating room gowns showed a bacterial penetration of 48.6%^37^ (Table 3).

**TABLE 3:** Bacterial Penetration Through SMs and CFCs

	SMs	
Reference	Study	Findings
Bowen^36^	Nonhuman	Filtration efficacy of 31.1% for bacterial-size particles with SM
Davies et al.^2^	Human	Filtration efficacy of 97.5% for SM against *Bacillus atrophaeus*
	**CFCs**	
Guyton et al.^35^	Human	Filtration efficacies of 28%-73% for CFC against *Bacillus globii* aerosols
Bowen^36^	Nonhuman	Filtration efficacy of 11.3% for CFC (bandana) against bacterial-size particles
Davies et al.^2^	Human	Filtration efficacy of 60%-83.2% against *Bacillus atrophaeus*

#### d) Source Control

Although a primary function of SMs and CFCs is serving as a barrier to exhaled respiratory particles (source control), research data addressing this topic are relatively scarce. A mannequin study using a mechanical cough machine reported that a SMs captured roughly 20% of exhaled aerosols during coughing 20 cm away from a second mannequin that wore no mask. The same study reported that source control during coughing, using a SM or N95 respirator was statistically superior to the same protection on the receiver, whereas during tidal breathing source control was comparable or superior to protection for the receiver.^38^

Experiments by Davies et al.^2^ showed that both SMs and CFCs reduced the mean microorganisms expelled by volunteers when coughing, with SMs being more effective at decreasing the number of microorganisms expelled than CFCs, especially at lower particle sizes. Overall, the CFCs was very tolerable, but functioned only one-third as well as a SM. A human study of 37 volunteers noted a 3.4-fold decrease in influenza viral copy numbers of large and small droplets expelled during breathing with SMs.^39^ Hui et al.^40^ used a human patient simulator in a supine position and reported that, during a simulated cough, the exhaled air dispersion distances were 68, 30, and 15 cm with no mask, SM, and N95 respirator, respectively. A study of 246 adults and children,^41^ investigating the impact of wearing SMs on shedding of seasonal coronavirus, rhinovirus, and influenza virus during breathing and coughing, reported a significantly reduced detection of influenza virus RNA in respiratory droplets and coronavirus RNA in aerosols, with a trend toward reduced detection of coronavirus RNA in respiratory droplets. A recent small study of coronavirus disease 2019 (COVID-19) patients,^42^ with baseline viral load of 5.66 log copies/mL, reported 2.56, 2.42, and 1.85 log copies/mL in Petri dishes placed 20 cm away from coughing patients who wore no mask, a SM, and cloth facemask, respectively (Table 4).

**TABLE 4:** Source Control Studies of SMs and CFCs

	SMs	
Reference	Study Type	Results
Patel et al.^38^	Mannequin aerosol study	SM capture of radiolabeled particles ~5%-20% during tidal breathing and ~35%-40% during coughing
Davies et al.^2^	Human study (21 subjects)	SM 89.5% capture of virus (23 nm) during coughing (*P* < **0**.001)
Milton et al.^39^	Human study (37 subjects)	SM 3.4-fold reduction in influenza **RNA** copies during tidal breathing **and** coughing (***P*** < .001)
Hui et al.^40^	Patient simulator (seated)	Sagittal and lateral expelled air dispersions during coughing with an **SM**were 30 cm and 28 cm compared **with** 68 cm and 0 cm without a SM
Leung et al.^41^	Randomized human study	Significantly reduced detection of (246 adults and children) influenza RNA in respiratory droplets and coronavirus in respiratory **aerosols** with SM
Bae et al.^42^	Human study (4 patients	During coughing, median viral loads with COVID-19 disease) were 2.56 and 2.42 log copies/mL, respectively, without and with a SM
	**CFCs**	
Davies et al.^2^	Human study (21 subjects)	CFC 48%-72.4% capture of virus (23 nm) during coughing
Bae et al.^42^	Human study (4 patients	During coughing, median viral loads with COVID-19 disease) were 2.56 and 1.85 log copies/mL, **with** and without a CFC

## DISCUSSSION

Scientific research data on the protective value of CFCs are very limited and precludes indepth interpretations of their value as a public health measure. Available data suggest that the protective factor of tested CFC generally is approximately half that of a SM, but variability exists (Table 1). Nonetheless, inasmuch as a protective factor >1 indicates some protection^27^ and CFCs have shown protective factors ranging from 1.8 to 3.2 (Table 1), their use offers some potential benefit. Given that the ability to prevent leakage into SMs or CFCs is primarily related to their seal to the face, limited available fit factor data suggest that some fabrics used to make CFCs could offer better facial sealing qualities than some models of SMs.^2^ For example, it has been shown that an overlay of a layer of nylon hosiery can significantly enhance the fit of CFCs or SMs.^43^ Other techniques, such as use of a layer of petroleum jelly (Vaseline),^37^ or taping,^25^ at the face/interface of SM or CFC may also enhance fit. Additionally, one cannot overestimate the potential impact of the type of tethering device (eg, encircling ties, elasticized straps, elasticized ear loops)^2^ and the presence or absence of a moldable nasal bar upon securing a good fit of SMs, CFCs, and respirators.

Viral penetrations through SMs and CFCs (Table 2) are variable, based upon fabric features (eg, weave, thickness, porosity) and flow rates used for testing. However, on average, CFCs tend to perform at a level ≤ half that of SM regarding viral penetration. The larger dimensions of bacteria, in comparison with viruses (Table 3), make them more amenable to capture by some CFCs, but, as with viral penetration, their performance is dependent upon fabric factors. Recent research has demonstrated that the best textiles for CFC construction are fabric hybrids (cotton/silk, cotton/chiffon, cotton/flannel) in multiple layers because of their filtration efficiencies and the added potential benefit of particle attraction and capture by means of their electorstatic properties.^44^ Other textile advances, such as a newly developed washable, electrostatic cotton that has been developed in South Korea and can serve as a filter insert into CFCs,^43^ will be important in determining the optimum fabrics for CFC construction. However, regardless of the filtration capabilities of the materials used for fabricating CFCs, it is the snugness of the fit of CFCs to the face that also will have a major impact on its protective abilities.

Inasmuch as CFCs cannot be considered respirators, source control remains a hoped-for primary feature in risk reduction. Although some decrease in viral shedding by humans through the use of CFCs has been reported,^2^ and decreased dispersal distances during cough have been demonstrated for barrier protection,^40^ the limited data available preclude a definitive overall assessment of the value of this intervention (Table 4). An optical study,^45^ using schlieren photography, indicates that coughing results in massive air leakage around the top and sides of SMs, so that other factors are also important in source control (eg, distance between individuals, air exchanges within rooms, air dilution outdoors). Nonetheless, the fact that CFCs have been shown to have some positive effect as source control (Table 4), however limited, would seem to make it intuitive to promote their use.

There are several objective and subjective benefits from wearing CFCs. In the context of the unavailability of adequate respiratory protective equipment, as with the current SARS-CoV-2 pandemic, having some protection may be better than nothing at all,^2,27,42^ while accepting that CFCs will generally not function as well as SMs or respirators. However, the CFC must be well constructed and used appropriately within an accepted framework that incudes frequent handwashing, ensuring that the mask covers the nose, mouth, and chin regions, avoidance of touching the outer aspect of the mask with the hands, snug application of the tethering devices (eg, straps, elastic ties) to avoid gaps between the face and CFC thereby improving fit, change out of CFCs when damp or soiled with body fluids, daily washing and drying of CFCs, removal from behind the head using the posterior aspects of tethering devices for grasping (followed by handwashing), and no sharing of the CFCs with others.^46^ In addition to some (limited) infection control potential, CFCs may serve to highlight not touching the face, the promotion of civic duty, and serve as a reminder of the need for social distancing. HCPs using CFCs should concurrently wear a face shield,^47^ if available, that addresses eye protection and provides additional protection from exhaled large droplets and splashes, sprays, and spatter of body fluids.^48^

Negative aspects of CFC use are limited protection, improper use and handling that may help spread infection, inducement of a false sense of security, loss of facial clues in communication, and some impairment in speech clarity. Other negative CFC issues include their lack of standards testing, poor fit that leads to increased manipulation of the CFCs and increased chance for infection, poor face sealing characteristics allowing for greater leaks, possibly some difficulty with breathing and liquid diffusion of infectious agents through damp CFCs.^33,49,50^ Studies addressing physiological responses to SMs and N95 respirator wear at HCP work rates over 1-2 h have shown that, in general, respiratory, cardiovascular, and thermal (core temperature) responses are mild,^51-54^ and this would likely apply to CFCs (depending on fabric factors). It should be emphasized that, for some individuals (eg, poorly controlled asthmatics, persons with a history of panic attacks or claustrophobia), wearing of CFCs may be problematic.

Perhaps the most important, unresolved issue of the use of CFCs and SMs is their actual impact on the public in terms of disease transmission. Large population-based studies on this theme are inherently difficult to accomplish because of numerous confounding variables (vaccination rates, compliance, quarantine mandates, etc.). A recent review of smaller studies of facemask use^55^ in community settings (4 studies of 143 to 617 households and 2 university student studies of 1178 and 1437 students), that generally also incorporated hand hygiene, found that hand hygiene alone was not effective against viruses, thereby suggesting that facemasks were the protective component. A recent large population-based study^56^ from Hong Kong (population 7.45 million) with facemask compliance estimated at 95%-97% based on periodic surveillance of all 18 of its administrative districts, reported that the incidence of COVID-19 cases during the first 100 d of the coronavirus outbreak was significantly lower (126 cases per million population) than similarly geographically sized and populated Singapore (259.8 cases per million population), where the government initially discouraged the public from wearing facemasks to conserve them for HCPs. This is indeed encouraging evidence, but needs to be reinforced by further studies.

Guidelines from the World Health Organization (WHO) during the coronavirus pandemic are that SMs and CFCs should not be worn by the general public, but rather by HCPs and caregivers of infected persons as protection from large aerosols and body fluids, by infected patients as a source control, and by others who are actively sneezing and coughing (could be an otherwise asymptomatic infected individual).^57^ The European Centre for Disease Prevention and Control has released guidance on the use CFCs by HCPs when SMs and respirators are unavailable, stating that they are inferior to SMs and should be used only as a last resort.^50^ The recent concern over the spread of SARS-CoV-2 by asymptomatic carriers has prompted the CDC to recommend the use of CFCs by the general populace in public settings where social distancing may be difficult.^1^ The compulsory use of CFCs in some Asian (eg, China) and European countries (eg, Austria, Czech Republic, Slovakia)^58^ indicates that this measure will become more commonplace throughout the world now, and possibly with other infectious outbreaks. Guidelines continue to develop as experience with the SARS-CoV-2 increases, but increased scientific research into the use and value of CFC is clearly warranted.

Strengths of the current study are that it reports on a timely topic of significant public health importance and offers a synopsis of the scientifically determined data on the protective capabilities of CFC. Limitations of the current report are that the literature search used only 2 search engines and was limited to English language articles. Also, the limited number of scientific articles precludes a definitive analysis of the overall value of CFCs in pandemic situations.

## CONCLUSIONS

In the face of the SARS-CoV-2 pandemic, the issue of personal protective equipment has become of paramount importance. Recommendations for the general public use of CFCs have not been made on an empirical basis, but rather on the assumption that (hopefully) some benefit can be attained. The available, but relatively scant, laboratory data and rare human research data on the use of CFCs tend to suggest some limited value to their use as barrier protection, but generally this benefit will be half or less than that of SMs and significantly less than that of N95 respirators. The use of CFCs is not without potential risks and must be used in conjunction with other infection control measures. CFCs are a low tier of infection control measures, the value of which is currently uncertain, and should be used only when other respiratory protective equipment is scarce or unavailable, while cognizant of their limitations. Research is sorely needed on the impact of CFCs on the respiratory transmission of infectious agents.

## References

[1] Centers for Disease Control and Prevention. Coronavirus disease 2019 (COVID-19). Recommendation regarding the use of cloth face coverings, especially in areas of significant community-based transmission. https://www.cdc.gov/coronavirus/2019-ncov/prevent-getting-sick/cloth-face-cover.html. Published April 3, 2020. Accessed April 4, 2020.

[2] Davies A , Thompson K-A , Giri K , et al. Testing the efficacy of homemade masks: would they protect in an influenza pandemic? Disaster Med Pub Health Preparedness. 2013;7:413-418. doi: 10.1017/dmp.2013.43 PMC7108646 24229526

[3] Mikulicz-Radecki J. Das operieren in steriliserten zwirnhandschuren und mit Mundbind. Zentralbl Chir. 1897;24:713-717.

[4] Flugge C. Die verreitung der phthise durch staubtormiges sputurm und durch beim husten Verspritzte tropfchen. Z Hyg Infectionshkr. 1898;29:107-124.

[5] Blair J , Herron T , Kuht JA , et al. Do theatre staff use face masks in accordance with the manufacturers’ guidelines of use. J Infect Prevent. 2019;20:99-106. doi: 10.1177/1757177418815551 PMC6437337 30944594

[6] Hamilton A. Dissemination of streptococci through invisible sputum: in relation to scarlet fever and sepsis. JAMA. 1905;44:1108-1111. https://jamanetwork.com/journals/jama/article-abstract/465640. Accessed April 3, 2020. doi: 10.1001/jama.1905.92500410032001g

[7] Capps JA. A new adaptation of the face mask in control of contagious disease. JAMA. 1918;70:910-911. https://jamanetwork.com/journals/jama/article-abstract/217690. Accessed March 2, 2020. doi: 10.1001/jama.1918.26010130001009 PMC10210761 37756982

[8] Schrader ES. From apron to gown: a history of OR attire. AORN J. 1976;24:52-67. doi: 10.1016/s0001-2092(07)64629-8 779640

[9] Yassi A, Bryce E. Protecting the faces of health care workers: knowledge gaps and research priorities for effective protection from occupationally-acquired respiratory infectious diseases. Occupational Health and Safety Agency for Healthcare in British Columbia, 2004. Semantic Scholar web site. https://www.paho.org/hq/dmdocuments/2009/Protecting%20the%20faces%20of%20health%20care%20workers.pdf. Published May 30, 2008. Accessed March 27, 2020.

[10] Centers for Disease Control and Prevention. The National Personal Protective Technology Laboratory (NPPTL). Respirator trusted-source information. https://www.cdc.gov/niosh/npptl/topics/respirators/disp_part/respsource3healthcare.html. Published May 25, 2018. Accessed June 3, 2020.

[11] US Food and Drug Administration. N95 respirators and surgical masks (face masks). https://www.fda.gov/medical-devices/personal-protective-equipment-infection-control/n95-respirators-and-surgical-masks-face-masks. Published December 16, 2019. Accessed March 27, 2020.

[12] US Food and Drug Administration. Surgical masks – premarket notification [510k] submissions. Guidance for industry and FDA staff. https://www.fda.gov/regulatory-information/search-fda-guidance-documents/surgical-masks-premarket-notification-510k-submissions. Published March 5, 2004. Accessed March 28, 2020.

[13] Vincent M , Edwards P. Disposable surgical facemasks for preventing surgical wound infection in clean surgery. Cochrane Database Syst Rev. 2016;4(4):CD002929. doi: 10.1002/14651858.CD002929.pub3 PMC7138271 27115326

[14] Rengasamy S , Shaffer R , Williams B , et al. A comparison of facemask and respirator filtration test methods, J Occup Environ Hyg. 2017;14:92-103. doi: 10.1080/15459624.2016.1225157 27540979 PMC7157953

[15] Rockwood CA Jr , O’Donoghue DH. The surgical mask: its development, usage and efficiency. A review of the literature, and new experimental studies. Arch Surg. 1960;80:963-971. doi: 10.1001/archsurg.1960.01290230081010 14438122

[16] Lynteris C. Plague masks: the visual emergence of anti-epidemic personal protection equipment. J Med Anthropol. 2018;37:442-457. doi: 10.1080/01459740.2017.1423072 30427733

[17] Chugtai AA , Seale H , MacIntyre CR. Use of cloth masks in the practice of infection control – evidence and policy gaps. Int J Infect Control. 2013;9:1-12. https://doi: 10.3396/IJIC.v9i3.020.13.

[18] Rengasamy S , Eimer B , Shaffer RE. Simple respiratory protection – evaluation of the filtration performance of cloth masks and common fabric materials against 20–1000 nm size particles. Ann Occup Hyg. 2010;54:789-798. doi: 10.1093/annhyg/meq044 20584862 PMC7314261

[19] Vanderbilt University Medical Center. Coronavirus (COVID-19) information for employees and patients. https://www.vumc.org/coronavirus/how-donate-hand-sewn-face-masks. Accessed March 30, 2020.

[20] Minnesota Department of Health. Interim guidance on alternative facemasks, 2020. https://www.health.state.mn.us/diseases/coronavirus/hcp/masksalt.pdf. Accessed March 31, 2020.

[21] Good Housekeeping Institute. Sachs L. How to make facemasks for hospitals during the coronavirus shortage. https://www.goodhousekeeping.com/health/a31902442/how-to-make-medical-face-masks/. Accessed March 31, 2020.

[22] Haller DA , Colwell RC. The protective quality of gauze face masks. JAMA. 1918;71:1213-1215. doi: 10.1001/jama.1918.26020410008008a

[23] US Department of Labor, Occupational Safety and Health Administration. Fit testing procedures. https://www.osha.gov/laws-regs/regulations/standardnumber/1910/1910.134AppA. Accessed March 27, 2020.

[24] Harriman KH , Brosseau LM. Respiratory protection for healthcare workers. Medscape Infectious Diseases 2011. https://www.medscape.com/viewarticle/741245_5. Accessed March 28, 2020.

[25] Kournikakis B , Harding RK , Tremblay JRA , et al. Comparison of protection factors for selected medical, industrial and military masks. J Am Biol Safety Assoc. 2000;5:12-18. doi: 10.1177/109135050000500105

[26] Lee S-A , Grinshpun SA , Reponen T. Respiratory performance offered by N95 respirators and surgical masks: human subject evaluation NaCl aerosol representing bacterial and viral size particle range. Ann Occup Hyg. 2008;52:177-185. doi: 10.1093/annhyg/men005 18326870 PMC7539566

[27] Van der Sande M , Teunis P , Sabel R. Professional and homemade face masks reduce exposure to respiratory infections among the general population. PLoS One. 2008;3(7):e2618. 18612429 10.1371/journal.pone.0002618 PMC2440799

[28] Gawn J , Clayton M , Makison C , et al. Evaluating the protection afforded by surgical masks against influenza bioaerosols. Health and Safety Executive/Health and Safety Laboratory, 2008. https://www.hse.gov.uk/research/rrpdf/rr619.pdf. Accessed March 27, 2020.

[29] Dato VM , Hostler D , Hahn ME. Simple respiratory mask. Emerg Infect Dis. 2006;12:1033-1034. doi: 10.3201/eid1206.051468 16752475 PMC3373043

[30] Oberg T , Brosseau LM. Surical mask fit and filter performance. Am J Infect Control. 2008;36:276-282. doi: 10.1016/j.ajic.2007.07.008 18455048 PMC7115281

[31] Balazy A , Toivola M , Adhikari A , et al. Do N95 respirators provide 95% protection level against airborne viruses, and how adequate are surgical masks? Am J Infect Control. 2006;34:51-57. doi: 10.1016/j.ajic.2005.08.018 16490606

[32] Shakya KM , Noyes A , Kallin R , et al. Evaluating the efficacy of cloth facemasks in reducing particulate matter exposure. J Expo Sci Environ Epidemiol. 2017;27:352-357. doi: 10.1038/jes.2016.42 27531371

[33] MacIntyre CR , Seale H , Dung TC , et al. A cluster randomized trial of cloth masks compared with medical masks in healthcare workers. BMJ Open. 2015;5:e006577. doi: 10.1136/bmjopen-2014-006577 PMC4420971 25903751

[34] Jung H , Kim J , Lee S , et al. Comparison of filtration efficacy and pressure drop in anti-yellow sand masks, quarantine masks, medical masks, general masks, and handkerchiefs. Aerosol Air Qual Res. 2014(4);991-1002. doi: 10.4209/aaqr.2013.06.0201

[35] Guyton HG , Decker HM , Anton GT. Emergency respiratory protection against radiological and biological aerosols. AMA Arch Ind Health. 1959;2:91-95. 13669760

[36] Bowen LE. Does that face mask really protect you? Appl Biosaf. 2010;15:67-71. doi: 10.1177/153567601001500204

[37] Ransjö U , Hambraeus A. An instrument for measuring bacterial penetration through fabrics used for barrier clothing. J Hyg. 1979;82:361-368. doi: 10.1017/s0022172400053894. 376694 PMC2130088

[38] Patel RB , Skaria SD , Mansour MM , et al. Respiratory source control using a surgical mask: an in vitro study. J Occup Environ Hyg. 2016:13:569-576. doi: 10.1080/15459624.2015.1043050 26225807 PMC4873718

[39] Milton DK , Fabian MP , Cowling BJ , et al. Influenza virus aerosols in human exhaled breath: particle size, culturability, and effect of surgical masks. PLoS Pathog. 2013;9(3):e1003205. 23505369 10.1371/journal.ppat.1003205 PMC3591312

[40] Hui DS , Chow BK , Chu L , et al. Exhaled air dispersion during coughing with and without a surgical or N95 mask. PLoS One. 2012;7(12):e50845. doi: 10.1371/journal.pone.0050845 23239991 PMC3516468

[41] Leung NHL , Chu DKW , Shiu EYC , et al. Respiratory virus shedding in exhaled breath and efficacy of face masks. Nat Med. 2020;26(5):676-680. doi: 10.1038/s41591-020-0843-2 32371934 PMC8238571

[42] Chugtai AA , Seale H , Macintyre CR. Effectiveness of cloth masks for protection against severe acute respiratory syndrome Coronavirus 2. Emerging Inf Dis. 2020;26: https://wwwnc.cdc.gov/eid/article/26/10/20-0948_article. Accessed November 6, 2020. 10.3201/eid2610.200948 PMC7510705 32639930

[43] Garcia Godoy LR , Jones AE , Anderson TN , et al. Facial protection for healthcare workers during pandemics: a scoping review. BMJ Glob Health. 2020;5:e002553. doi: 10.1136/bmjgh-2020-002553 PMC7228486 32371574

[44] Konda A , Prakash A , Moss GA , et al. Aerosol filtration efficiencies of common fabrics used in respiratory cloth masks. ACS Nano. 2020;14:6339-6347. doi: 10.1021/acsnano.0c03252 32329337

[45] Tang JW , Liebner TJ , Craven BA , et al. A schlieren optical study of the human cough with and without masks for aerosol infection control. J R Soc Interface. 2009;6(Suppl 6):S727-S736. 19815575 10.1098/rsif.2009.0295.focus PMC2843945

[46] Centers for Disease Control and Prevention. How to wear cloth face coverings. https://www.cdc.gov/coronavirus/2019-ncov/prevent-getting-sick/how-to-wear-cloth-face-coverings.html. Published May 23, 2020, Accessed June 1, 2020.

[47] Centers for Disease Control and Prevention. Strategies for optimizing the supply of facemasks. https://www.cdc.gov/coronavirus/2019-ncov/hcp/ppe-strategy/face-masks.html. Published March 17, 2020. Accessed April 6, 2020.

[48] Roberge RJ. Face shields for infection control: a review. J Occup Environ Hyg. 2016;13:235-242. doi: 10.1080/15459624.2015.1095302 26558413 PMC5015006

[49] Government of Canada. Considerations in the use of homemade masks to protect against COVID-19. Published with revision June 3, 2020. https://www.canada.ca/en/health-canada/services/drugs-health-products/medical-devices/activities/announcements/covid19-notice-home-made-masks.html. Accessed March 30, 2020.

[50] European Centre for Disease Prevention and Control. Cloth masks and mask sterilization as options in case of shortage of surgical masks and respirators. https://www.ecdc.europa.eu/sites/default/files/documents/Cloth-face-masks-in-case-shortage-surgical-masks-respirators2020-03-26.pdf. Published March 26, 2020. Accessed March 31, 2020.

[51] Kim J-H , Roberge RJ , Powell JB. Effect of external airflow resistive load on postural and exercise-associated cardiovascular and pulmonary responses in pregnancy. BMC Pregnancy Childbirth. 2015;15:45. doi: 10.1186/s12884-015-0474-7 25886031 PMC4357216

[52] Roberge RJ , Kim J-H , Benson SM. Absence of consequential changes in physiological, thermal and subjective responses from wearing a surgical mask. Respir Physiol Neurobiol. 2012;181:29-35. doi: 10.1016/j.resp.2012.01.010 22326638

[53] Roberge RJ , Coca A , Williams WJ , et al. Physiological impact of the N95 filtering facepiece respirator on healthcare workers. Resp Care. 2010;55:569-677. 20420727

[54] Roberge RJ , Kim J-H , Coca A. Protective facemask effect on human thermoregulation: an overview. Ann Occup Hyg. 2012;5:102-112. doi: 10.1093/annhyg/mer069 21917820

[55] MacIntyre CR , Chugtai AA. A rapid systematic review of the efficacy of facemasks and respirators against coronaviruses and other transmissible viruses for the community, healthcare workers and sick patients. Int J Nurs Stud. 2020;108:103629. doi: 10.1016/j.ijnurstu.2020.103629 32512240 PMC7191274

[56] Cheng VC-C , Wong S-C , Chuang VW-M , et al. The role of community-wide wearing of face mask for control of coronavirus disease 2019 (COVID-19) epidemic due to SARS-CoV-2). J Infect. 2020;81(1):107-114. doi: 10.1016/j.jinf.2020.04.025 32335167 PMC7177146

[57] World Health Organization. Coronavirus (COVID-19) advice for the public: when and how to use masks. https://www.who.int/emergencies/diseases/novel-coronavirus-2019/advice-for-public/when-and-how-to-use-masks. Published March 11, 2020. Accessed March 31, 2020.

[58] National Public Radio. In “public adjustment,” some European countries push for residents to wear masks. https://www.npr.org/sections/coronavirus-live-updates/2020/04/01/82580019/in-big-adjustment-some-european-countries-push-for-residents-to-wear-masks. Accessed April 4, 2020.

